# Urgent Use of Sugammadex in a Patient With Occult Bronchopleural Fistula Manifested Following Anesthesia Induction

**DOI:** 10.7759/cureus.84431

**Published:** 2025-05-19

**Authors:** Takashi Suzuki, Mizue Ishii, Reina Sakazaki, Daiki Nagane, Akiko Ogaku

**Affiliations:** 1 Department of Anesthesiology, Showa Medical University Koto Toyosu Hospital, Tokyo, JPN; 2 Department of Anesthesia and Intensive Care Medicine, National Cancer Center Hospital, Tokyo, JPN

**Keywords:** air leak, apneic oxygenation, diffusion oxygenation, empyema, occult bronchopleural fistula, one-lung ventilation, open window thoracostomy, oxygen insufflation, sugammadex, urgent use

## Abstract

Sugammadex could play a significant role in managing the “cannot intubate, cannot ventilate” scenarios. However, the urgent use of sugammadex in intubated patients after induction of anesthesia is uncommon. We report the case of an 85-year-old, 45 kg man with a history of pulmonary resection for lung cancer and open window thoracostomy for postoperative pyothorax. He underwent a ureteral stent exchange for ureteral calculi. During anesthesia induction, 40 mg of rocuronium was administered, followed by uneventful mask ventilation and tracheal intubation. Subsequent mechanical ventilation failed due to massive air leakage from an occult bronchopleural fistula in the left chest wall that manifested following anesthesia induction. However, oxygenation was maintained with oxygen-air insufflation from the anesthesia machine by closing the adjustable pressure-limiting valve and maximizing fresh gas flow. Although the tracheal tube was blindly advanced with the intention of one-lung ventilation via the right lung, it was unsuccessful. Subsequently, 200 mg of sugammadex was administered to reverse the rocuronium effect, allowing the continuation of anesthesia with desflurane under spontaneous breathing. High-flow oxygen-air insufflation via a tracheal tube and urgent, but not critical, use of sugammadex to restore spontaneous breathing were helpful for the anesthetic management of this patient who developed a massive air leak due to an occult bronchopleural fistula that manifested following anesthesia induction.

## Introduction

Although the usefulness of sugammadex in the critical situation of “cannot intubate, cannot ventilate” (CICV) during anesthesia induction remains controversial [1,2], there are numerous reports of its use as a rescue agent in such situations [2]. However, urgent use of sugammadex in already intubated patients after induction of anesthesia is uncommon. We describe a patient who had previously undergone open window thoracostomy (OWT) for the treatment of postoperative pyothorax following pulmonary resection for lung cancer, in whom positive pressure ventilation was jeopardized by a massive air leak caused by an unrecognized bronchopleural fistula (BPF) that emerged after the induction of anesthesia and initiation of mechanical ventilation. In this case, sugammadex restored spontaneous breathing, allowing anesthesia to continue. Written consent was obtained from the patient for publication of this report.

## Case presentation

**Figure 1:**
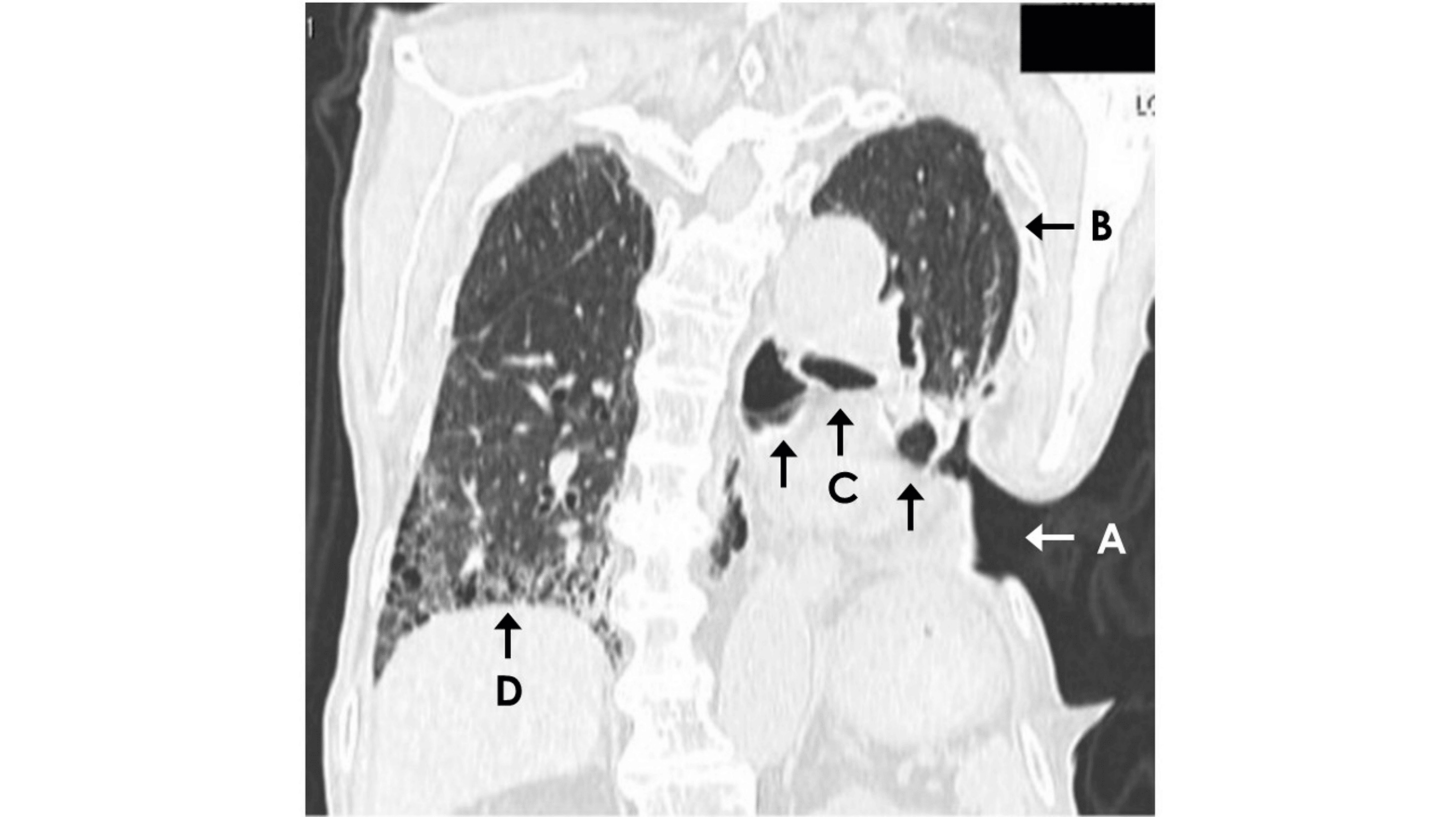
Coronal computed tomography scan of the chest Coronal chest computed tomography scan, performed one month after the presented anesthesia case, revealed an open window thoracostomy on the left posterior chest wall (arrow A) and the residual left upper lobe following surgical resection of the left lung (arrow B). The scan also showed multiple large bullae in the left upper lung lobe (arrows C) and a honeycomb lung pattern in the right lower lobe (arrow D).

An 85-year-old, 45-kg man with a history of lung cancer surgery, cholecystectomy, and chronic kidney disease was scheduled to undergo ureteral stent exchange for ureteral calculi. Preoperative laboratory tests revealed a serum potassium level of 5.8 mEq/L, serum creatinine of 2.16 mg/dL, and blood urea nitrogen of 48.3 mg/dL, with no other notable abnormalities. Since a previous stent placement under local anesthesia performed eight months earlier was a painful and frustrating experience for the patient, general anesthesia was chosen based on the patient's preference for the procedure. Although we did not obtain referral information from the hospital at which the patient had undergone lung surgery nearly two years earlier, he had undergone a left lower lobectomy. Moreover, the presence of a fenestration defect due to costectomy on the left posterior chest wall indicated that his postoperative course had been complicated by empyema. The visceral pleura could be seen at the bottom of the cavity through the oval-shaped fenestration measuring 9 cm by 7 cm. The cavity had been packed with dry gauze to protect the pleura, although there was a slight but persistent serous discharge. Hence, the dressing was changed daily during his current hospital stay. The preoperative pulmonary function tests were inconclusive due to a lack of cooperation. Although preoperative computed tomography of the chest was not performed, the image taken one month after this urological procedure showed marked interstitial changes in the lung and multiple large bullae (Figure 1).

On arrival in the operating room, his blood pressure was 166/93 mmHg, his heart rate was 63 bpm, and his arterial oxygen saturation by pulse oximetry (SpO₂) was 97% on room air. Neuromuscular monitoring was not applied. After preoxygenation, anesthesia was induced using 50 mcg of fentanyl, a continuous infusion of remifentanil at 0.1 mcg/kg/min, and 50 mg of propofol. Following confirmation that manual ventilation with a bag was feasible, 40 mg of rocuronium was administered. Adequate mask ventilation was confirmed by visible chest rise, and tracheal intubation with a 7.5 mm internal diameter tube was successfully performed without problems under videolaryngoscopic guidance by a trainee. Mechanical ventilation with a volume control mode, positive end-expiratory pressure of 5 cmH₂O, fresh gas flow (FGF) of 2 l/min, and an inspired oxygen concentration of 40% was started after several breaths of manual ventilation with 100% oxygen. Since the patient's blood pressure decreased to 66/46 mmHg immediately following intubation, remifentanil was discontinued, and 8 mg of intravenous ephedrine was administered. After achieving normotension, 1% desflurane inhalation was commenced. Mechanical ventilation was continued, although his ventilatory volume gradually decreased, and ventilation became almost ineffective several minutes after intubation, accompanied by collapse of the ventilator bellows. Hence, the FGF was increased to 15 l/min while maintaining the same inspired oxygen concentration. However, since this was ineffective in restoring ventilation, manual ventilation was attempted, which was also unsuccessful in providing actual ventilation since the reservoir bag remained only partially filled despite closing the adjustable pressure limiting valve. At this time, we continued insufflation of an oxygen-air mixture through the tracheal tube to maintain oxygenation. When we performed an exploratory check of the orifice of the cavity in the chest wall to investigate the source of the massive gas leak, we sensed airflow from the bottom of the cavity, which was augmented by the more significant inspiratory flow generated with activation of the oxygen flush valve of the anesthesia machine. At this stage, we recognized for the first time that the patient had an occult BPF in the left lung that had become manifest with continuous positive pressure ventilation, although its precise locus was unknown. Therefore, we blindly advanced the tracheal tube, which had been correctly placed, toward the right main bronchus in an attempt to establish one-lung ventilation (OLV) of the right lung. However, repeated attempts at proper tube placement for OLV were unsuccessful.

We, therefore, adjusted treatment strategies to establish ventilation and reverse neuromuscular blockade to restore spontaneous breathing. We removed the tracheal tube and inserted a #3 iGel® (Intersurgical Ltd., Wokingham, UK). This was followed by the administration of 200 mg sugammadex and discontinuation of desflurane. By this time, over 10 minutes had passed since the administration of rocuronium. Despite an insufflated oxygen concentration of 40%, SpO₂ was maintained at 100%. Therefore, we refrained from administering additional sugammadex and continued monitoring the patient. Spontaneous breathing resumed within a few minutes. Subsequently, 2% desflurane inhalation was initiated with an FGF of 4 l/min to begin the scheduled urological procedure. Several minutes after the start of surgery, the inspired desflurane concentration was increased to 3%. In this way, anesthesia was maintained under spontaneous breathing throughout the 15-minute urological procedure. The iGel® was removed 13 minutes after the end of surgery without any additional administration of sugammadex. SpO_2_ remained at 100% throughout anesthesia. The patient’s postoperative course was uneventful, and he was discharged on postoperative day two without any complications.

## Discussion

BPF, which results in leakage of inspired air from the airways into the pleural space, is a rare but potentially life-threatening complication of pulmonary resection [3]. BPFs are classified as either central or peripheral types based on the location of the air leak [4], with this case falling into the latter category. These fistulas are sometimes unrecognized due to minimal symptoms, as observed in our patient. Such cases are referred to as occult BPFs [5,6].

The fenestration in our patient’s chest wall was presumed to have been deliberately created through rib resection as part of the OWT, a long-established therapeutic option for long-term open drainage of postoperative empyema [7]. Due to limited information about the postoperative course of his pulmonary resection, we assumed that the OWT was performed as a therapeutic measure for empyema resulting from his lung surgery. During the preparation of this report, we confirmed that the OWT had indeed been performed two months after his lower lobe lobectomy due to the development of empyema. Generally, once definitive drainage of postoperative empyema is achieved with OWT, its closure is typically planned. However, in some cases, as with our patient, the closure either fails or is abandoned [8]. In this case, positive pressure ventilation was initially achievable during the induction of anesthesia but later became impossible. This likely occurred because continuous positive pressure ventilation reopened previously collapsed small airways, leading to the manifestation of the air leak from the occult BPF. Several options were considered to address the unexpected massive air leak from the left lung. While OLV of the right lung using a blindly advanced tracheal tube was initially considered a straightforward and prompt solution, the tube could not be positioned appropriately for this purpose for unknown reasons. Establishing OLV with a double-lumen endobronchial tube or a bronchial blocker under fiberoptic bronchoscopic guidance, though effective, was deemed too time-consuming, and the necessary equipment was not immediately available. Given that the proposed urological procedure was minimally invasive and unlikely to be lengthy, deep anesthesia was deemed unnecessary. Hence, as a second-line strategy, we successfully restored spontaneous breathing using sugammadex. To the best of our knowledge, this is the first reported use of sugammadex for the rapid restoration of spontaneous breathing in paralyzed and intubated patients with massive air leaks.

The dose of sugammadex administered in this case was 4.4 mg/kg, which is substantially lower than the recommended 16 mg/kg dose for “CICV” situations [2]. A recent systematic review of case reports on sugammadex use in “CICV” scenarios reported median rocuronium and sugammadex doses of 0.6 mg/kg and 14 mg/kg, respectively, with a median time interval between their administrations of six minutes. In contrast, the present case involved a higher rocuronium dose, a markedly lower sugammadex dose, and a substantially longer interval of over 10 minutes. This extended interval might partially account for the modest sugammadex dose required for restoring spontaneous breathing. Although the authors do not recommend administering sugammadex at a low dose in a CICV scenario, the review noted that successful rescue was achieved in half of the reported cases with doses lower than recommended [2]. A dose-response study has been conducted to evaluate the antagonistic effect of sugammadex on profound neuromuscular blockade induced by rocuronium [9]. According to the results, when 4 mg/kg of sugammadex was administered three minutes after a 1 mg/kg dose of rocuronium, the median time for the train-of-four ratio to reach 0.7, which is the level at which spontaneous breathing is generally expected to return but not safe for extubation, was 4.2 minutes, with a range of 2.6 to 7.1 minutes. In the present case, although neuromuscular monitoring was not performed, the doses of rocuronium and sugammadex were 0.9 mg/kg and 4.4 mg/kg, respectively, which are comparable to those in the aforementioned study. Moreover, sugammadex was given approximately 10 minutes after the administration of rocuronium. Therefore, the return of spontaneous breathing within a few minutes following sugammadex administration cannot be considered an unusual outcome under these conditions. If performing the surgery under spontaneous breathing had proven difficult, the authors would have administered an additional dose of sugammadex.

It has long been known that oxygenation and carbon dioxide removal can be achieved in apneic and paralyzed patients by oxygen insufflation into the central airway [10]. This phenomenon is referred to as apneic oxygenation, diffusion oxygenation, apneic ventilation, or similar terms. For instance, a constant oxygen flow of 0.6 to 0.7 L/min into both main bronchi via thin catheters inserted orally has been reported to maintain oxygenation in the clinical setting [11]. In the present case, SpO_2_ was maintained at 100% by continuous insufflation of 15 l/min of 40% oxygen above the carina after positive pressure ventilation failed due to a massive air leak. Therefore, we administered sugammadex in an urgent manner; however, it was not used in a ‘cannot oxygenate’ situation. It may be considered an ironic phenomenon, but the presence of a massive air leak may have enabled effective apneic oxygenation using a high-flow oxygen-air mixture via the tracheal tube, which likely promoted both oxygenation and carbon dioxide elimination.

In retrospect, the anesthetic management of this case warrants discussion. First, medical history-taking and preoperative patient assessment were insufficient. If preoperative chest computed tomography had been performed, prior identification of the multiple large bullae might have allowed for more thorough preparation to anticipate and manage air leaks during anesthesia. Second, there was a lack of awareness and understanding regarding the occult BPF. When applying conventional ventilation in patients with BPFs, it is recommended to minimize the development of air leaks by reducing tidal volume, lowering positive end-expiratory pressure, shortening inspiratory time, decreasing the respiratory rate, and accepting permissive hypercapnia [12]. Third, and this is the simplest and most obvious consideration, neuraxial anesthesia should have been chosen instead of general anesthesia after adequate explanation to the patient.

## Conclusions

We report a case of an unexpected and abrupt massive air leak that jeopardized positive pressure ventilation following anesthesia induction and intubation in a patient who had previously undergone OWT and had occult BPF. However, oxygenation was maintained using high-flow mixed oxygen-air insufflation through the tracheal tube via an anesthesia machine. Furthermore, although the situation was not critical, we believed that the urgent administration of sugammadex to promote the restoration of spontaneous breathing was instrumental in successfully managing this uncommon case of ventilatory failure.
